# Long-Term Follow-Up of Adamantinoma of the Tibia Complicated by Metastases and a Second Unrelated Primary Cancer: A Case Report and Literature Review

**DOI:** 10.1155/2018/5493750

**Published:** 2018-03-25

**Authors:** Brendan R. Southam, Alvin H. Crawford, David A. Billmire, James Geller, Daniel Von Allmen, Adam P. Schumaier, Sara Szabo

**Affiliations:** ^1^Department of Orthopaedics and Sports Medicine, University of Cincinnati, Cincinnati, OH 45220, USA; ^2^Department of Orthopaedics, Cincinnati Children's Hospital Medical Center, Cincinnati, OH 45229, USA; ^3^Department of Plastic Surgery, Cincinnati Children's Hospital Medical Center, Cincinnati, OH 45229, USA; ^4^Department of Oncology, Cincinnati Children's Hospital Medical Center, Cincinnati, OH 45229, USA; ^5^Department of Surgery, Cincinnati Children's Hospital Medical Center, Cincinnati, OH 45229, USA; ^6^Department of Pathology, Cincinnati Children's Hospital Medical Center, Cincinnati, OH 45229, USA

## Abstract

Adamantinoma is a rare, low-grade malignant tumor of the bone which grows slowly and typically occurs in the diaphysis of long bones, particularly in the tibia. Adamantinomas have the potential for local recurrence and may metastasize to the lungs, lymph nodes, or bone. We report a case of a 14-year-old female with a tibial adamantinoma who underwent wide resection with limb salvage and has subsequently been followed up for 18 years. The patient went on to have both a local soft tissue recurrence 5 years after the resection and metastases to both an inguinal lymph node and the right lower lobe of the lung 8 years after that recurrence, all of which have been treated successfully with marginal resections. Unique to this case, the patient was also incidentally found to have chromophobe-type renal cell carcinoma when undergoing a partial nephrectomy to resect a presumed metastasis of her adamantinoma. Genetic testing has not revealed any known genetic predisposition to cancer.

## 1. Introduction

Adamantinomas are rare, accounting for less than 1% of primary malignancies of the bone [1–4]. Classically, adamantinomas occur in the long bones, particularly in the tibia in up to 80 to 90% of patients, and have a predilection for the mid-diaphyseal region [3, 5–7]. Involvement of a synchronous lesion in the ipsilateral fibula has been reported in approximately 10% of cases [5, 8, 9]. Adamantinomas occur most frequently in adolescents and young adults with a mean age of 25–35 years [2, 5, 9–11]. Previous series have identified a slight male predominance [8, 12]. Given the indolent nature of this malignancy, it typically has a long, progressive clinical course characterized by swelling, pain, and deformity prior to diagnosis [3, 5, 9].

Radiographically, the tumor tends to be eccentric, expansile, and osteolytic with a sharply or poorly defined sclerotic margin [5, 9, 13]. Not infrequently, multiple lytic foci are present with surrounding sclerosis giving the lesion a so-called “soap bubble appearance” [3, 5, 9, 13]. Significant anterior cortical disruption of the tibia is common and may be present with extension of the lesion into the medullary canal or the surrounding soft tissues resulting in anterior bowing [5, 9, 11]. MRI has proven crucial for determining the amount of intramedullary and soft tissue involvement of these tumors, assisting in preoperative planning [10, 14–17]. CT may also be useful for assessing cortical destruction and detecting subtle pathologic fractures, present in up to 23% of patients [10, 18].

Histologically, adamantinomas are low-grade biphasic malignant tumors, with a typically nesting or cord-like epithelial component in a bland osteofibrous stroma. Considerable variability in the relative amounts of these two components may be observed. In the well-differentiated variant, an osteofibrous dysplasia-like stromal component predominates, with small-to-inconspicuous epithelial nests and peripheral woven bone spicules that are rimmed by osteoblasts [3, 5, 7, 9]. This variant more commonly affects patients in the first two decades of life. The typical presentation of tumors in adults and metastatic lesions is the classic variant; in the classic variant, the epithelial component predominates, and its patterns may be described as spindle, squamous, basaloid, and tubular type [3, 5, 10, 19]. Another intermediate histological variant osteofibrous dysplasia- (OFD-) like adamantinoma has also been recognized in the literature. Similar to OFD, this subtype presents typically within the first two decades of life, producing an intracortical lesion of the tibia [9, 10, 20]. Histologically, it is characterized by a similar stroma to OFD with small scant nests of epithelial cells detectable with light microscopy [9, 20, 21]. The other top differential diagnoses to be considered based on histology and location are osteofibrous dysplasia and adamantinoma-like Ewing sarcoma.

Adamantinomas were historically thought to be localized malignancies with limited metastatic potential given their typically indolent course [22]. However, more recent literature has demonstrated relatively high rates of local recurrence and metastases, particularly following an incomplete resection. Given the relative rarity of these tumors, most of the literature has been limited to case series with inadequate long-term follow-up, making it challenging to determine definitive treatment guidelines for these patients. Chemotherapy and radiation have largely proven ineffective, and amputation was historically considered the mainstay of treatment [9, 10, 12, 23–26]. More recently, wide en bloc resection with subsequent reconstruction has proven highly effective for limb preservation with good functional outcomes and equivocal rates of recurrence, metastases, and survival [5, 12, 18, 23, 27, 28]. A variety of options for limb reconstruction exist including intercalary allografts, vascularized and nonvascularized fibular autografts, distraction osteogenesis, and segmental metallic implants [18, 27, 29–32]. Intercalary allografts, however, have emerged as the preferred form of reconstruction in the literature [3, 18].

We report a case of a tibial adamantinoma that was treated with a limb salvage procedure utilizing an intercalary tibial allograft and a free vascularized osteocutaneous graft of the fibula. The patient later had a local recurrence treated with a secondary salvage procedure and subsequent metastases to the lung and an inguinal lymph node treated with marginal resections. A second rare primary cancer, chromophobe-type renal cell carcinoma, was later discovered incidentally in this patient, despite no known genetic predisposition. A review of the literature is provided.

## 2. Case Study

**Figure 1:**
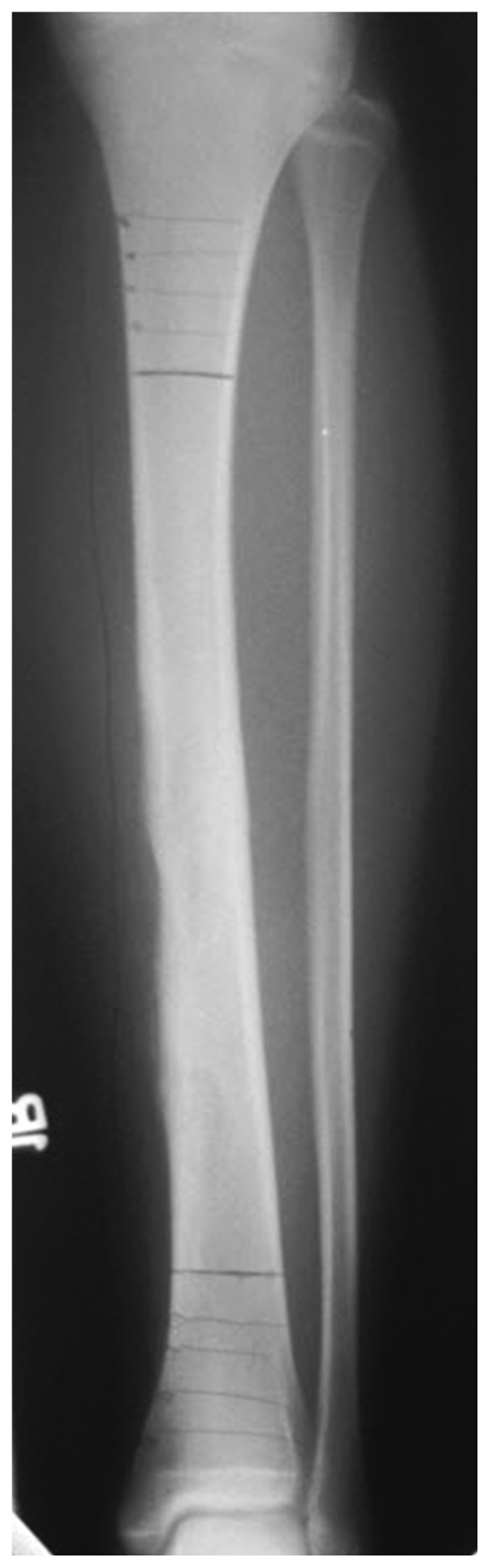
An anteroposterior radiograph of the right tibia and fibula obtained following curettage and bone grafting demonstrating a predominantly cortical-based expansile lesion involving the distal diaphysis consistent with adamantinoma.

A 14-year-old Caucasian female presented initially to an outside provider with a mass in the right midshin which had grown slowly in size over the course of two years causing increasing discomfort. Initial conservative management included observation and rest. Due to an acute exacerbation of pain, radiographs of the right tibia and fibula were obtained demonstrating a predominantly cortical-based lobulated lesion of the distal tibial diaphysis with overlying soft tissue irregularity (Figure 1). The patient underwent curettage and bone grafting of the lesion. Permanent pathology sections revealed an adamantinoma with epithelial cells in tubular and squamous patterns which had diffuse CD99 reactivity and cytokeratin-positive staining [3, 5, 10, 33]. This prompted referral to our institution for definitive treatment. The patient's past medical history was unremarkable, and family history of cancer was only notable for prostate cancer in a paternal grandfather.

**Figure 2:**
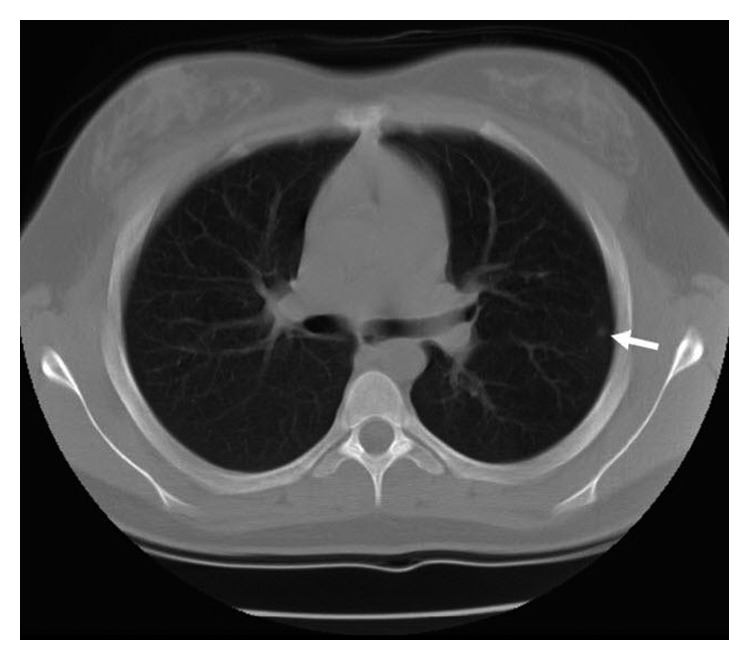
An axial computed tomography (CT) scan of the chest demonstrating a subpleural lesion (white arrow) identified at the time the adamantinoma was originally diagnosed. The lesion was biopsied due to concerns of metastasis, and the biopsy revealed benign fibrous lung tissue.

A metastatic workup including a bone scan revealed increased uptake in the midtibia consistent with the previously diagnosed tumor. Additionally, a CT scan of the chest revealed several small nodules in the left upper lobe of the lung which were felt to be consistent with metastatic disease; however, an open biopsy demonstrated only evidence of chronic pleuritis with fibrosis (Figure 2).

**Figure 3:**
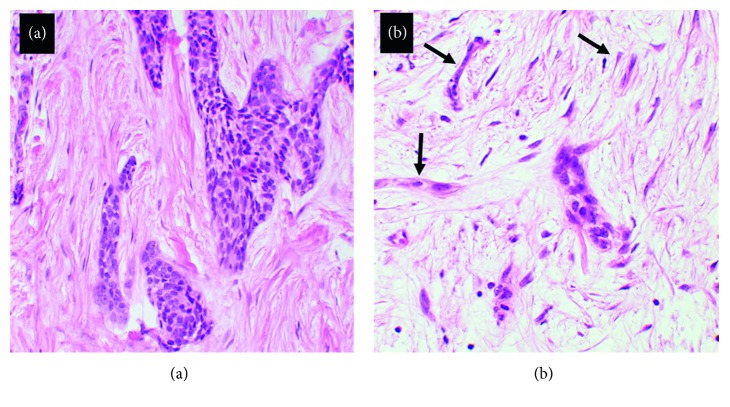
(a) Well-differentiated variants of adamantinoma including cords and islands of epithelial cells with a basaloid appearance, with some squamoid differentiation, were observed in this patient. (b) In other areas sampled, double rows of epithelial cells impart a tubular appearance or occur in single-cell cords (black arrows).

**Figure 4:**
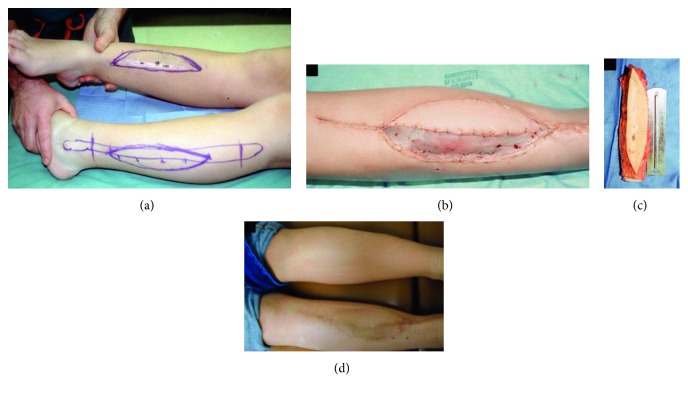
(a) A clinical image demonstrating the planned resection margins of the right lower extremity prior to the limb salvage procedure. The osteocutaneous flap from the contralateral extremity has also been marked out. (b) A postoperative image demonstrates that the medial osteocutaneous flap is well perfused. (c) Osteocutaneous free vascularized fibula flap. (d) The flap went on to incorporate well.

**Figure 5:**
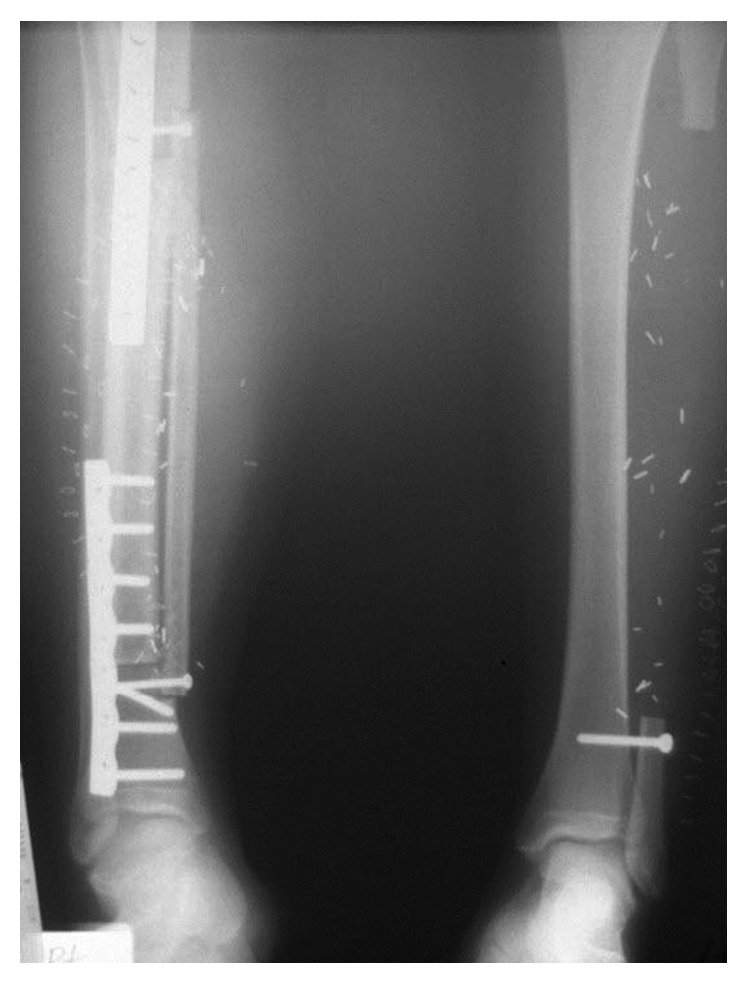
An anteroposterior radiograph of the bilateral lower legs following adamantinoma resection and limb reconstruction. On the right, the allograft is positioned laterally with the vascularized fibular graft placed overlying it medially. The grafts are fixed by plates and screws. On the left, the fibula has been resected and the ankle mortise has been stabilized proximally with a screw.

After thorough preoperative counseling, the patient elected to undergo resection of the adamantinoma with limb salvage. A 19 cm section of the medial tibial shaft was resected along with a 20 × 5 cm section of the overlying skin and subcutaneous tissue down to the level of the anteromedial fascia, as this was assumed to have been contaminated from the original biopsy (Figure 3). The defect was then partially reconstructed laterally with an intercalary tibial allograft which was fixed both proximally and distally. Prior to placing the graft, the proximal metaphyseal tibia had been curetted for the bone graft which was placed into the intramedullary canal adjacent to the allograft. Simultaneously, plastic surgeons elevated an osteocutaneous fibular flap from the lateral aspect of the left leg. The free vascularized osteocutaneous graft of the fibula was placed to fill the remaining defect of the right medial tibia adjacent to the previously placed allograft (Figures 4 and 5). End-to-end and end-to-side anastomoses were performed with the microsurgical technique between the peroneal and posterior tibial veins and arteries, respectively. A tibialis anterior muscle flap was used for coverage of the allograft on the reconstructed right tibia. On the left donor-site side, a syndesmotic screw was placed to fix the distal fibula. Skin grafts from the left lateral thigh were obtained to provide coverage of the remaining skin defects on both legs. Frozen sections reviewed intraoperatively and permanent sections all confirmed negative margins. The patient was made non-weight-bearing after surgery to allow for graft consolidation.

**Figure 6:**
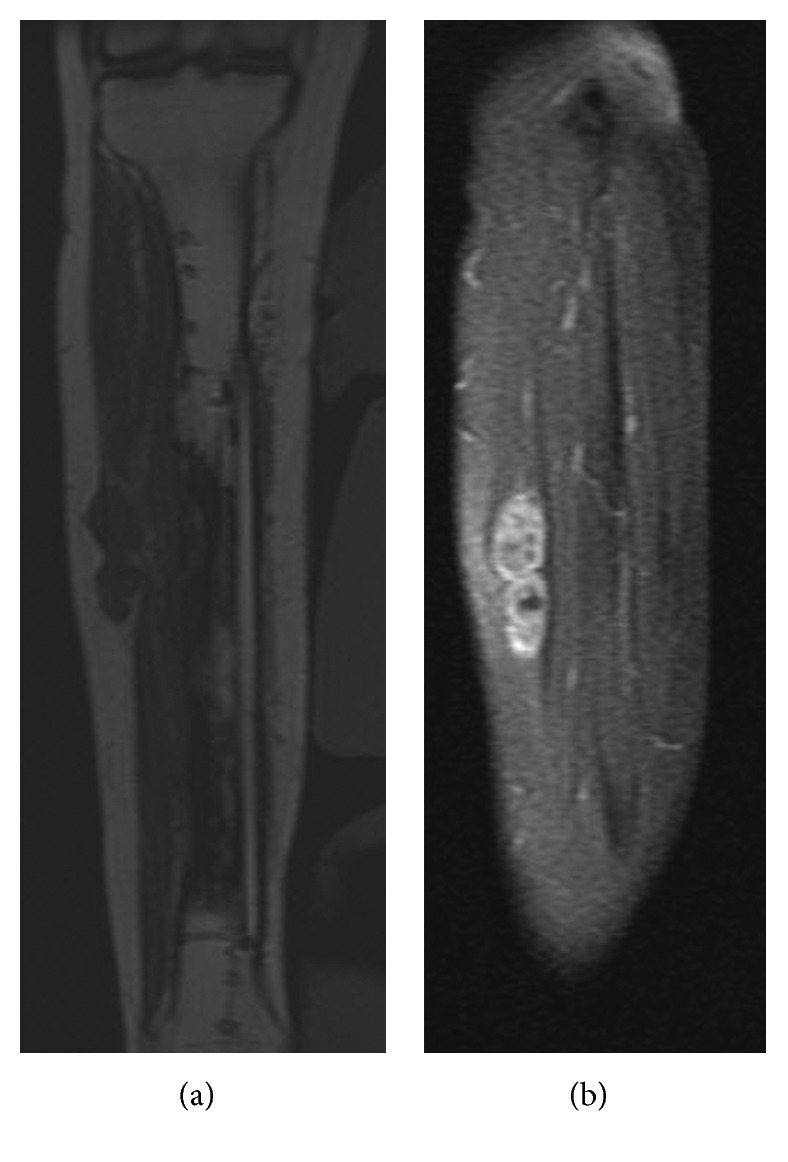
(a) A coronal T1-weighted magnetic resonance image showing a dumbbell-shaped mass in the anterolateral subcutaneous tissues involving the underlying extensor hallucis longus and extensor digitorum longus muscles. (b) The mass enhances diffusely after gadolinium administration, consistent with a soft tissue mass.

The patient had an uneventful recovery, and serial radiographs demonstrated slow incorporation of the allograft. She later developed a stress fracture of the graft two and a half years postoperatively that subsequently healed. Four years after the initial resection, the patient began to experience recurrent pain and swelling of the right lower extremity. Radiographs demonstrated medial and pretibial soft tissue swelling over the previous tibial reconstruction, but a CT scan failed to demonstrate any lesion. Following conservative management with ibuprofen 800 mg and a compressive wrap with symptomatic control, nine months later, she elected for removal of hardware as this was thought to potentially be the source of her pain. The patient subsequently developed palpable masses on the right lower leg several months after the hardware was removed. MRI demonstrated a dumbbell-shaped mass measuring 5.4 × 1.7 × 2.1 cm in size arising in the soft tissues of the anterolateral aspect of the shin involving both the extensor hallucis longus and extensor digitorum longus muscles (Figure 6). A positron emission tomography (PET) scan, bone scan, and contrasted CT of the chest abdomen and pelvis were obtained and were unremarkable for metastatic disease. An open biopsy was performed and confirmed the diagnosis of recurrent adamantinoma.

**Figure 7:**
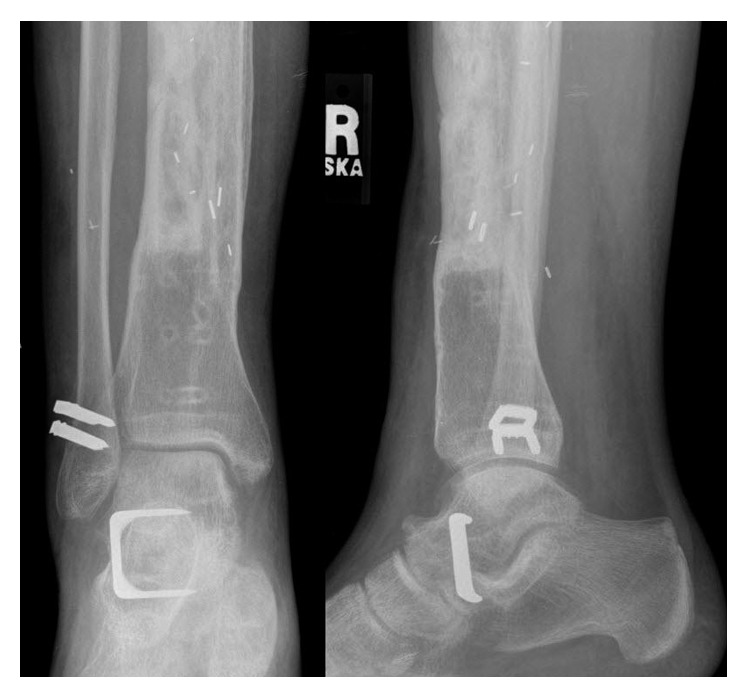
An AP and lateral view of the right ankle following a posterior-to-anterior tibialis tendon transfer, peroneal tenodesis, and subtalar fusion for an acquired foot drop resulting from resection of the soft tissue recurrence in the anterior compartment.

After multiple discussions with the family regarding amputation versus a repeat limb salvage procedure, the patient elected to undergo local resection. The entirety of the anterior compartment was resected leaving a 10 × 25 cm soft tissue defect which plastic surgery reconstructed using a free-flap transfer of the entire left rectus muscle. The inferior epigastric vessels were anastomosed with the microsurgical technique to the anterior tibial vessels, and a skin graft was obtained from the previous donor site to cover the remaining skin defect from the resection. All margins of the soft tissue resection were negative. The patient was left with a residual drop foot following the procedure and 6 months later underwent a subtalar arthroereisis and peroneal tenodesis along with a posterior tibialis tendon transfer to anterior tibialis tendon transfer (Figure 7). Following this period, the patient returned every 6 months for regular surveillance including radiographs and an MRI of the right lower extremity.

**Figure 8:**
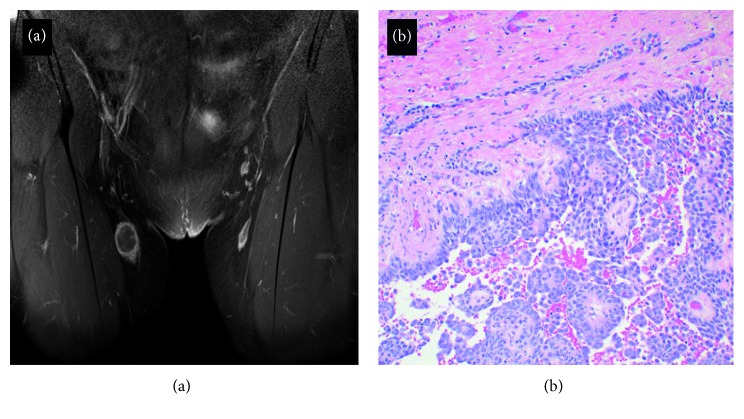
(a) A coronal fat-suppressed T1 image with gadolinium enhancement demonstrating a mass in the right groin with an irregular rim of peripheral enhancement. This mass was excised and found to be a metastasis of the adamantinoma to an inguinal lymph node. (b) The epithelial component of the metastasis regionally imparts a pseudopapillary appearance, with intervening hemorrhage.

**Figure 9:**
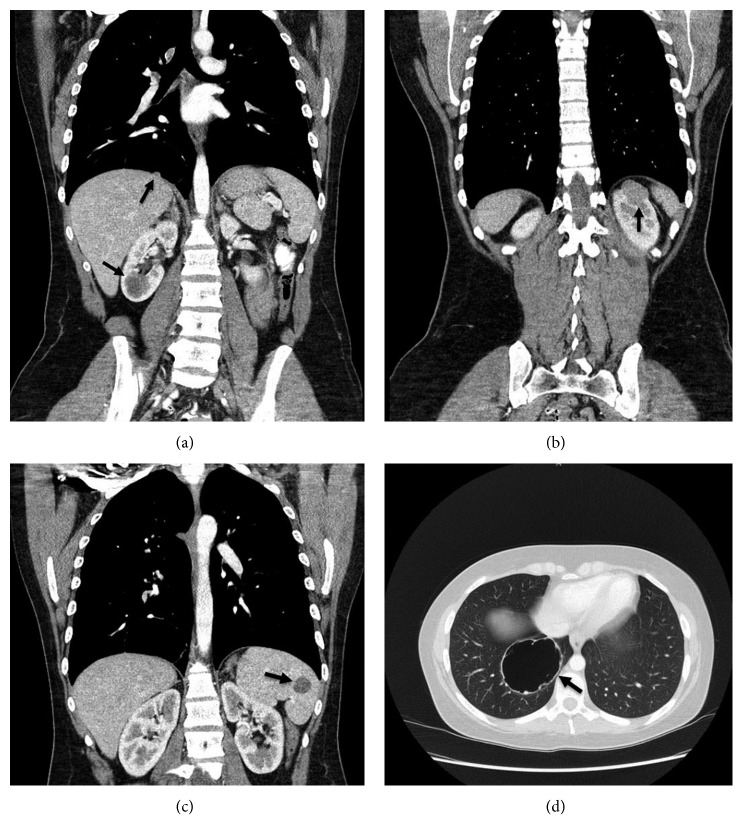
Computed tomography scans of the chest, abdomen, and pelvis demonstrating multiple lesions including a hypodense lesion of the spleen, bilateral kidney lesions, and a large cystic lesion with mural nodularity in the right lower lobe of the lung. The image on the top right demonstrates the lesion that was found to be chromophobe-type renal cell carcinoma in the upper pole of the left kidney, and the image on the bottom right shows the large metastatic adamantinoma lesion in the right lung.

At the age of 25, 11 years after initial presentation, the patient began to experience erythema and swelling of the skin graft on the right lower leg managed with oral antibiotics and subsequently by topical corticosteroids. The swelling resolved and was later assumed to be an allergic reaction from wearing compression stockings. At this time, she also noticed a mobile palpable mass in her right groin which bothered her only when she was exercising. This was felt to be an enlarged lymph node, and the decision was made to continue to follow the patient closely. Two years later, an MRI demonstrated a 2 cm round lesion in the right thigh with increased T2 signal and peripheral enhancement. A PET scan was also obtained which showed increased uptake in the right inguinal region. Thus, the patient underwent an excisional biopsy of the lymph node that revealed a metastasis of the adamantinoma (Figure 8). The patient subsequently had a CT of the chest, abdomen, and pelvis performed which revealed lesions in her bilateral kidneys and spleen and a large lesion in the right lower lobe of the lung (Figure 9).

**Figure 10:**
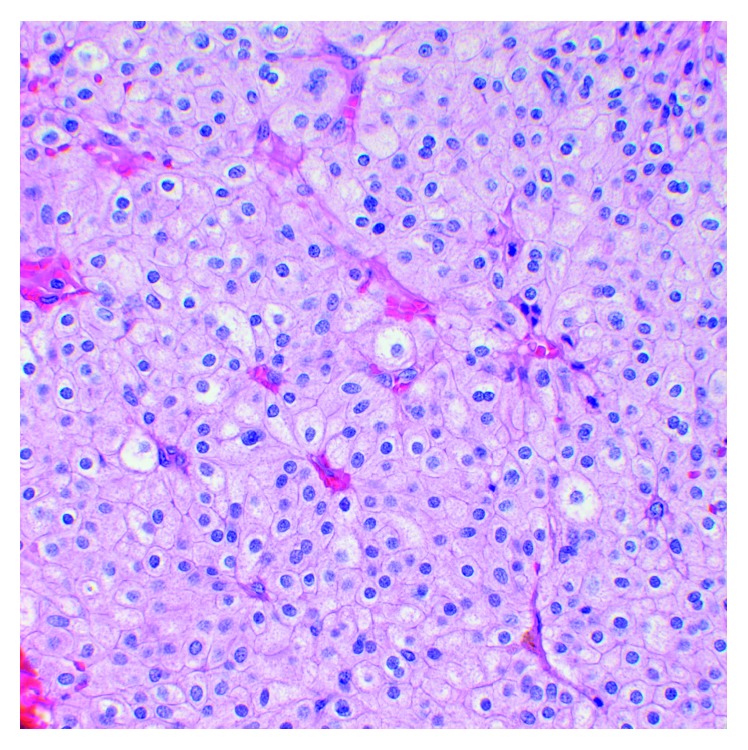
A hematoxylin and eosin stain demonstrating representative histomorphology of the incidentally diagnosed chromophobe-type renal cell carcinoma.

General surgery performed a right lower lobectomy via thoracotomy which also revealed metastatic disease. Two months later, the patient underwent a robot-assisted laparoscopic splenectomy and partial nephrectomies of both kidneys. Pathology revealed that the right kidney and splenic lesions were both benign cysts, but the left kidney was stage T1A chromophobe-type renal cell carcinoma (chRCC), a second primary cancer, with microscopic positive margins (Figure 10). Given the improved prognosis with this subtype of RCC and a lower risk for metastasis, the decision was made to monitor the patient closely with serial imaging at 3-month intervals [34]. The patient developed a postsplenectomy thrombocytosis that has been monitored closely, and she completed two years of penicillin VK for sepsis prophylaxis. She has had no recurrence of either cancer on surveillance imaging. Given the history of two primary cancers, the patient later elected to undergo genetic testing involving an 18-gene panel of known renal cancer genes and was found to have no known genetic predisposition. Four years after the final resections, the patient developed a small mass at the base of her right neck just superior to the shoulder blade. An MRI was obtained which demonstrated the mass with a thin capsule and a fatty signal. She elected to have the mass removed, and pathology revealed adipose tissue consistent with a lipoma.

## 3. Discussion

Although adamantinomas are low-grade malignancies, local recurrence and metastases are not uncommon. The most frequent sites of metastases are the lungs. Lymph node metastases occur less frequently, with rare metastases to the bone reported as well [8, 12, 14]. In the series by Moon and Mori, 21 patients had 29 sites of clinical metastases at the time of death: 16 of the sites were pulmonary and 5 were in the inguinal lymph nodes [12]. In a review by Keeney et al. of 85 cases of adamantinoma, 31% of patients had recurrent local disease, 15% developed lung metastases, and 7% developed lymph node metastases. Of those patients with lung metastases, 69% had a preceding local recurrence as was the case in this patient.

In a review of 28 patients with a diagnosed adamantinoma from the Netherlands Bone Tumor Registry, nine patients (32%) had a local recurrence after a mean 7-year follow-up. All of these patients had undergone an intralesional or marginal resection. Of these, three patients went on to have metastatic disease. An additional five patients without local recurrence also developed metastatic disease. However, no patients who had undergone an en bloc resection developed local recurrence or metastases. Thus, an overall rate of metastases of 29% was observed. Intralesional or marginal resection was found to be the most significant risk factor for a local recurrence or metastasis [23]. Other significant risk factors for recurrence included duration of symptoms less than one year, pain at the time of presentation, and an age less than twenty years at the time of diagnosis.

In a recent international multicenter retrospective review of 70 cases of adamantinoma, 91% of patients had a limb salvage procedure performed with roughly half the patients receiving an intercalary allograft reconstruction. The limb preservation rate at a median follow-up of 7 years was 84%. The rate of metastasis was 10%, and the rate of local recurrence at 10-year follow-up was 18.6%, with a 10-year survival rate of 87.2%. En bloc tumor resection with wide operative margins at the time of limb salvage was associated with a significantly lower risk of local recurrence. In patients who underwent reconstructions, nonunion and fracture were the most common complications, occurring in 24% and 23% of patients, respectively [18]. In this case, the patient experienced both delayed union and allograft fracture that subsequently healed.

Previously, the use of vascularized fibular grafts with massive allografts has been described in the literature [30, 35–37]. This well-established technique combines the mechanical strength of the allograft with the biologic potential of the fibular graft to revascularize and incorporate. This reduces the inherent risks associated with pure intercalary allograft reconstructions which include delayed unions, nonunions, and stress fractures as previously discussed, as well as an increased risk of infection [30, 35–38]. More recently, osteocutaneous fibular grafts used in combination with allografts have been described for the management of long bone defects. Halim et al. achieved favorable long-term outcomes in a series of 12 patients with a mean follow-up of 63 months who underwent this technique. They noted that osteocutaneous flaps provided soft tissue coverage at the time of reconstruction and allowed for a tensionless closure which did not compromise microvascular anastomoses. Three vascularized flaps in this series developed venous thrombosis recognized by the reduced Doppler signal and congestion of the skin paddle. This allowed for early detection and intervention leading to successful salvage of the flap in all cases [38].

Due to the slow-growing nature of adamantinomas, local recurrences and metastases have been documented in the literature up to 36 years after the original diagnosis [8, 9, 39–43]. Therefore, long-term surveillance (>15 years) is warranted in these patients as was highlighted by the late metastases observed in the present case [14, 23, 41, 44]. Furthermore, imaging of the lungs and physical examination for lymphadenopathy may be considered on an annual basis or at the time of local recurrence to monitor for metastases [10]. Metastases and recurrences discovered through routine surveillance should be managed by surgical resection [10, 42, 44].

In this patient, routine surveillance resulted in the incidental detection of a second primary malignancy which may have otherwise been missed. It also resulted in additional morbidity with the patient undergoing a partial nephrectomy, splenectomy, and a prior open biopsy of the lung, all of which revealed benign tissue. However, in light of this patient, we feel that suspected metastases to unusual locations outside of the lungs, lymph nodes, or bone should be evaluated carefully as these may represent other malignancies.

To the best of our knowledge, this is the only reported case in the literature of a second primary malignancy in a patient with adamantinoma [45]. There are no known germline mutations associated with adamantinoma. Cytogenetic analyses of 9 cases of adamantinoma and osteofibrous dysplasia have identified several trisomies which have potentially been implicated with the histogenesis of these lesions, including trisomies 7, 8, 12, 19, and 22 [46]. Interestingly, chRCC is also a rare malignancy accounting for roughly 5–10% of cases of diagnosed RCC, which translates to projected 3200–6400 cases in the United States in 2016 [47, 48]. Although most cases of chRCC are sporadic, some are associated with a familial form known as Birt–Hogg–Dubé syndrome which is caused by an inactivating mutation of the *FLCN* gene [48, 49]. However, this patient was found not to be a carrier of this mutation. Given multiple metachronous primary cancers in one patient, it is possible that she is a carrier of a genetic mutation that has not yet been identified which predisposes her to malignancy [50].

## 4. Conclusion

Adamantinoma is a rare, low-grade malignant tumor of the bone associated with an indolent course due to its slow growth. However, high rates of late local recurrence and metastases have been reported in the literature. En bloc resection with wide operative margins followed by limb reconstruction has now become the standard of care, producing good functional outcomes along with high rates of limb preservation and overall survival. It is imperative to continue to follow these patients for greater than 15 years to allow for early detection of recurrent disease or metastases. In the case of presumed metastases, consideration of other malignancies is warranted, particularly when these lesions are identified in areas uncommon for adamantinoma metastases.
